# Deoxynivalenol damages the intestinal barrier and biota of the broiler chickens

**DOI:** 10.1186/s12917-022-03392-4

**Published:** 2022-08-15

**Authors:** Shuangxiu Wan, Na Sun, Hongquan Li, Ajab Khan, Xiaozhong Zheng, Yaogui Sun, Ruiwen Fan

**Affiliations:** 1 Shanxi Key Lab. for Modernization of TCVM, College of Veterinary Medicine, Shanxi Agricultural University, Taigu, 030801 Shanxi China; 2 College of Pharmacy, Heze University, Heze, Shangdong 274000 People’s Republic of China; 3 Centre for Inflammation Research, Queen’s Medical Research Institute, The University of Edinburgh, Edinburgh, EH16 4TJ UK; 4 College of Veterinary Medicine, Shanxi Agricultural University, Taigu, Shanxi 030801 People’s Republic of China

**Keywords:** Broiler, Deoxynivalenol, Production performance, Intestinal biota, Tight junction protein

## Abstract

**Background:**

In the livestock feed industry, feed and feed raw materials are extremely susceptible to mycotoxin contamination. Deoxynivalenol (DON) is one of the main risk factors for mycotoxin contamination in broiler feed and feedstuff, however, there is still little knowledge about this. Hence, the purpose of this study was to explore the toxicity effect of DON on the intestinal barrier and the microecological balance of the biota in broiler chickens.

**Results:**

In our present study, we compared the pathological scores of the small intestines of broilers on the 5^th^, 7^th^, and 10^th^ day, and chose the 7^th^ day to analyze the small intestine histomorphology, tight junctions, and cecal biota of the broilers. The results showed the damage to the small intestine worsened over time, the small intestinal villi of broilers were breakage, the tight junctions of the small intestine were destroyed, the cecal biota was unbalanced, and the growth performance of broilers was reduced on the 7^th^ day.

**Conclusions:**

DON could damage the functional and structural completeness of the intestinal tract, disorder the Intestinal biota, and finally lead to declined broiler performance. Our study provided a basis for the prevention and treatment of DON in broiler production.

**Supplementary Information:**

The online version contains supplementary material available at 10.1186/s12917-022-03392-4.

## Background

In the livestock feed industry, feed and feed raw materials are extremely susceptible to mycotoxin contamination. Deoxynivalenol (DON) in the trichothecene group B, produced by *Fusarium graminearum* is one of the main risk factors of mycotoxin contamination in broiler feed and feedstuff [1]. DON is also known as deoxynivalenol (3a, 7a, 15-trihydroxyfusarium-9-en-8-one), and its toxic effect could be maintained for more than one year under natural conditions, even for four years [2]. The main toxic effects of DON are cytotoxicity [3, 4], immunotoxicity [5, 6], neurotoxicity [7, 8], and synergistic effects with other biological toxins [9]. According to the tests conducted by Alltech Laboratories (China), deoxynivalenol and zearalenone were two of the main source of mycotoxin contamination in animal feed in 2018. The analysis of 44 types of mycotoxins from 411 animal feed samples in 24 provinces, autonomous regions, and municipalities across the country revealed that the detection rate of fumonisins, trichothecenes B (deoxynivalenol), and zearalenone were all greater than 85% in 2019. Among the 149 feed and 34 litter samples in the first half of 2020, each sample was contaminated with 8.34 types of mycotoxins on average. The detection rates of fumonisins, trichothecenes B, and zearalenone were all greater than 92% (https://www.sohu.com/a/304707386_653825 and https://www.sohu.com/a/376490762_653825). Therefore, deoxynivalenol contamination of feed was still a problem that seriously affects broiler production.

Intestinal mucosal biological, mechanical, chemical, and immune barriers as well as the microecological balance of the intestinal biota are important for maintaining the healthy growth and production of animals. However, in recent years, there have been few studies on DON’s damage to the intestinal barrier of broiler chickens and the microecological balance of the biota [1, 10, 11]. The mechanism underlying the damage to the intestinal barrier, the changes in the intestinal microbiota, and the impact on the production performance of broilers by DON need to be elucidated. The purpose of this experiment was to study the toxic effects of DON on the growth performance, mechanical barrier of the small intestine, and the cecal biota of broilers which could provide a basis for the prevention and treatment of DON toxicity in broiler production.

## Results

### Intestinal pathology score

Compared with the control group, no significant difference was observed for the intestinal pathology scores of broilers on the 5^th^ day (*p* > 0.05). However, the intestinal scores of broilers increased significantly (*p* < 0.05) on both the 7^th^ day and 10^th^ day (Table 1), so the broilers on day 7 were chosen for the experiment.

**Table 1:** Results of pathological scores of intestinal damage caused by DON in broilers

Item	5 d		7 d		10 d	
	Control group	DON group	Control group	DON group	Control group	DON group
Visual score	0	0.38 ± 0.48	0.25 ± 0.43	1.75 ± 1.56^**^	0.13 ± 0.33	4.25 ± 0.83^***^
Histological score	0.002	0.25 ± 0.43	0.38 ± 0.41	2.75 ± 1.50^**^	0.25 ± 0.43	5.25 ± 1.50^***^

Data were expressed as the mean ± SEM

^**^p < 0.01

^***^p < 0.001

### The effect of DON on the growth performance of broilers

Compared with the control group, the ADG, ADFI, and F/G of broilers in the DON group were decreased, among which ADG and ADFI were decreased significantly (*p* < 0.05). The results showed that the consumption of DON contaminated feed reduced the growth performance of broilers (Table 2).

**Table 2:** The effects of DON on the growth performance of broilers

Items	Control group	DON group	SEM	P-value
ADG(g)	16.78***	12.98	0.824	0.0001
ADFI(g)	23.71**	20.15	1.253	0.0097
F/G	1.82	1.44	0.258	0.171

Abbreviation: ADG Average Daily Gain, ADFI Average Daily Feed Intake, F/G ratio of feed to gain

^**^p < 0.01

^***^p < 0.001

### Morphological changes of small intestinal villi

**Fig. 1:**
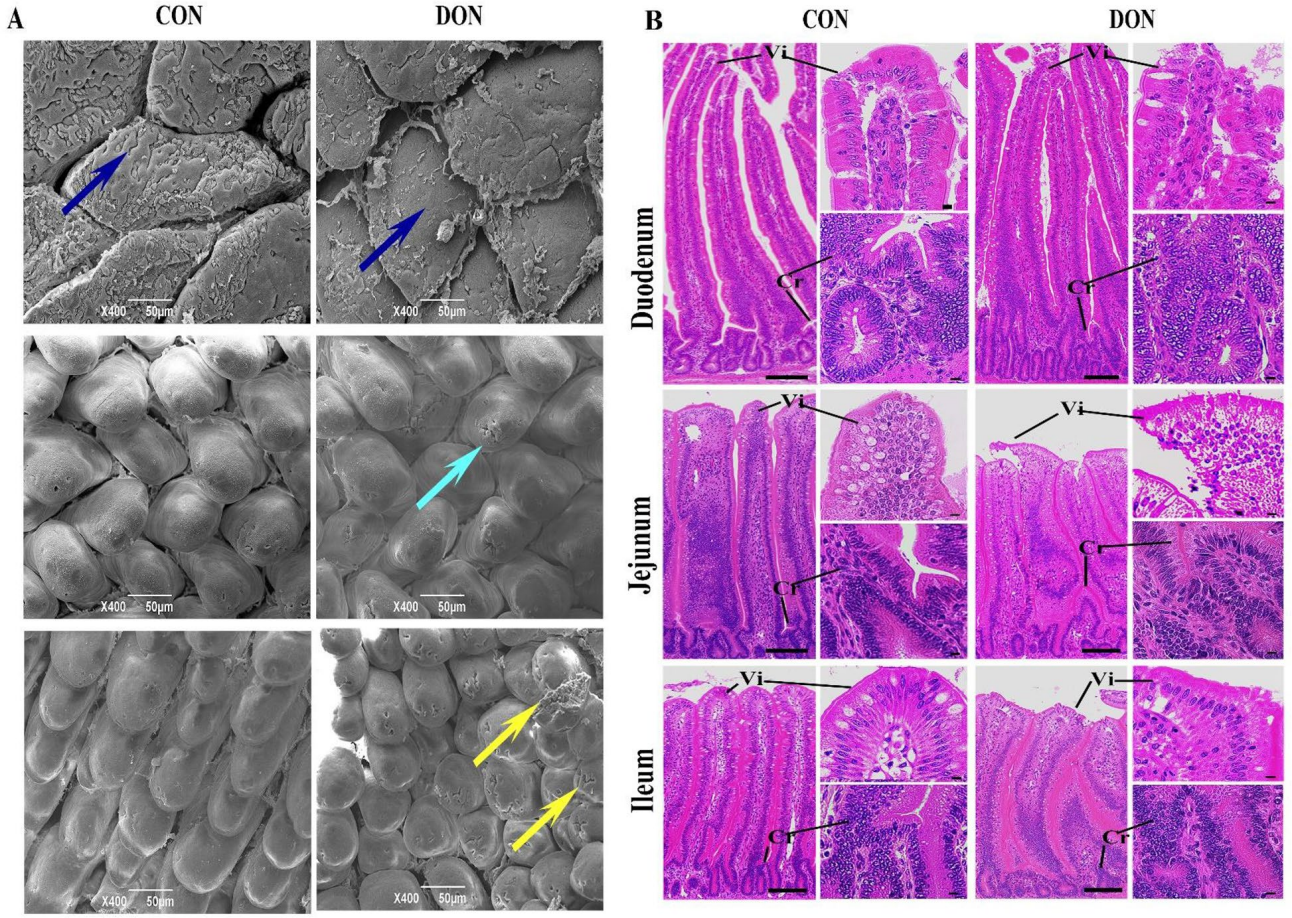
Morphology analysis of the small intestinal villi of broilers. **A** The scanning electron microscope of the duodenum, jejunum, and ileum in broilers. The Figure showed the pathological changes in the swollen duodenal mucosal surface with less chyme (blue arrow), ulcers and ulcer foci with different sizes in the jejunal surface (cyan arrow), and broken villi in the mucosal surface of the ilium (yellow arrow) of the DON group. Scale bar:50 μm. **B** H&E staining of the duodenum, jejunum, and ileum in broilers. The figure showed the damaged edges of duodenal villi, the loosely arranged outer cells, the increased crypt depth and the irregular ileum villi in the DON group. Vi: villus. Cr: crypt. Scale bar:100 μm

As shown in Fig. 1A, scanning electron microscopy analysis demonstrated that compared with the control group, the duodenal mucosal surface of the DON group possessed less chyme and was swollen (blue arrow). Ulcers of different sizes (cyan arrow) were observed in the jejunal surface and ulcer foci of different sizes. Broken villi (yellow arrow) were also observed in the mucosal surface of the ilium.

Compared with the control group, H&E staining demonstrated that the edges of duodenal villi in the DON group were damaged. The outer cells were loosely arranged. The villus height and the villi/crypt ratio were significantly reduced (*p* < 0.05). The crypt depth was significantly increased (*p* < 0.05). There was shedding in the jejunum. The villus height was significantly reduced (*p* < 0.05). The crypt depth and the ratio between villi and crypt were reduced, although not significantly. In ileum the villi were irregular, and their heights were different (not significantly). The crypt depth and the villi/crypt ratio were significantly reduced (*p* < 0.05) (Fig. 1B and Table 3).

**Table 3:** Effect of DON on intestinal pathology

Parameter	Control group	DON group	SEM	*P*-value
Duodenum				
Villus height (μm)	787.99 ± 31.57^***^	657.39 ± 50.50	19.850	*P* < 0.0001
Crypt depth (μm)	81.20 ± 16.64	124.02 ± 19.55^***^	8.558	*P* < 0.0001
Villus/crypt ratio	10.03 ± 1.65^***^	5.43 ± 0.92	0.630	*P* < 0.0001
Jejunum				
Villus height (μm)	540.62 ± 17.40***	462.19 ± 9.67	6.634	*P* < 0.0001
Crypt depth (μm)	70.77 ± 10.42	63.12 ± 10.17	4.854	0.132
Villus/crypt ratio	7.85 ± 1.45	7.49 ± 1.04	0.595	0.547
Ileum				
Villus height (μm)	441.29 ± 25.55	428.62 ± 10.32	9.186	0.185
Crypt depth (μm)	55.64 ± 10.99	66.47 ± 6.70*	4.289	0.021
Villus/crypt ratio	8.30 ± 1.97*	6.52 ± 0.72	0.700	0.020

Data were expressed as the mean ± SEM

^*^p < 0.05

^***^p < 0.001

Overall, the scanning electron microscopy and H&E staining revealed that the DON induced pathological damage, such as swollen tips of intestinal villi, damaged intestinal villi, and hyperplasia of intestinal crypts.

### Change in the digestive enzyme activities of the small intestine in broilers

Compared with the control group, the amylase, trypsin, and lipase activities in the DON treated group were reduced. The activity of amylase and trypsin in the duodenum were significantly reduced (*p*<0.05), the lipase activity was non-significantly different (*p*>0.05); the trypsin in the jejunum was significantly reduced (*p*<0.05), but the activity of amylase and lipase showed a decreasing trend (*p*>0.05); the amylase and trypsin activities in the ileum were significantly decreased (*p*<0.05), while that of lipase were non-significantly changed (*p*>0.05), as shown in Table 4.

**Table 4:** The digestive enzyme activities in each segment of the small intestine

Items	Control group	DON group	SEM	*P*-value
**Duodenum**				
Amylase U/mg prot	15.39*	14.97	0.201	0.0472
Trypsin U/mg prot	64.43***	63.57	0.179	*p* < 0.0001
Lipase U/g prot	3.01	2.96	0.0552	0.352
**Jejunum**				
Amylase U/mg prot	445.43	443.64	1.402	0.215
Trypsin U/mg prot	88.31**	87.37	0.300	0.0048
Lipase U/g prot	3.93	3.87	0.0433	0.225
**Ileum**				
Amylase U/mg prot	198.91*	195.58	1.267	0.0155
Trypsin U/mg prot	172.61**	171.88	0.207	0.0019
Lipase U/g prot	10.72	10.47	0.623	0.174

^*^p < 0.05

^**^p < 0.01

^***^p < 0.001

### Expression analysis of the tight junction proteins in the small intestine

**Fig. 2:**
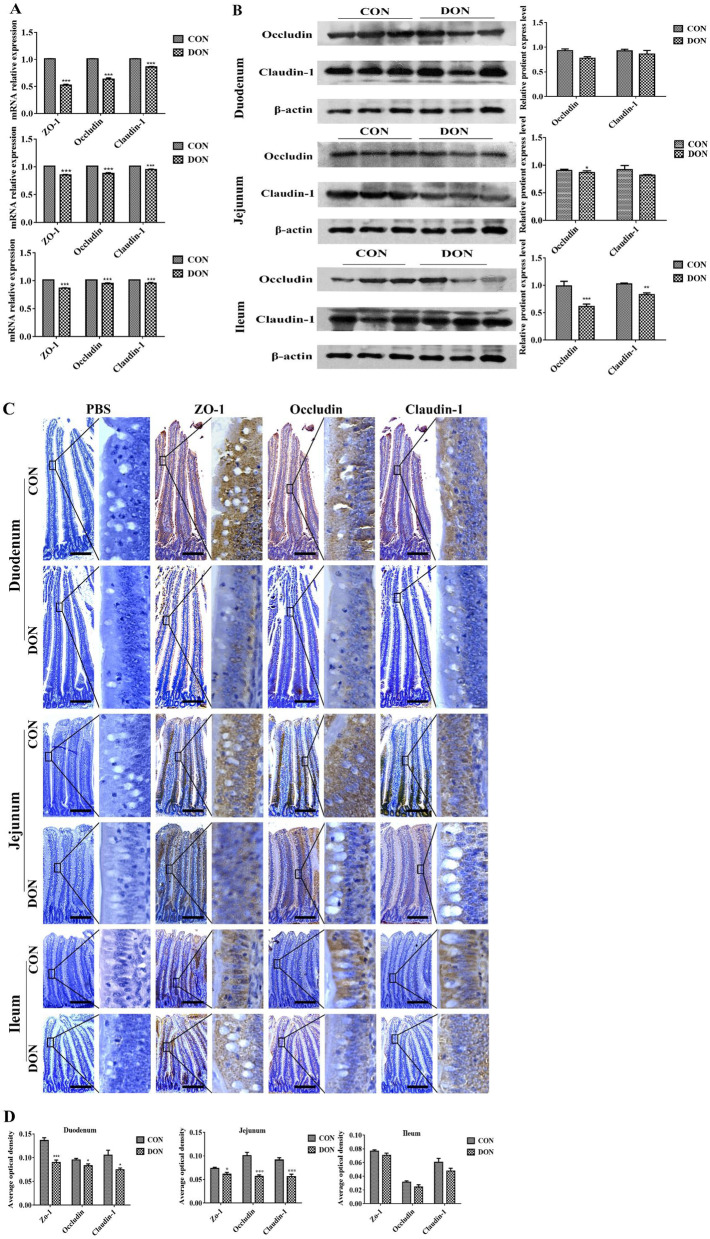
The effects of DON on the expression of tight junction proteins in the small intestine of broilers. **A** The mRNA relative expression of ZO-1, Occludin and claudin-1 analysed by qRT-PCR. **B** The relative expression of Occludin and claudin-1 protein detected by Western Blot. **C** Immunohistochemistry of the ZO-1, Occludin and Claudin-1. **D** The immunohistochemistry quantified results were showed as the average optical density. Scale bar:100 μm. The right image was magnified 1000 times, *n* = 6 per treatment group. *p < 0.05, **p < 0.01, ***p < 0.001. EC: Epithelial Cells

qRT-PCR was used to detect the mRNA expression of ZO-1, Occludin, and Claudin-1 in each segment of the small intestine. The relative mRNA expression of ZO-1, Occludin, and claudin-1 in the jejunum, ileum, and duodenum of the DON group were significantly lower than the control group (*p* < 0.05, Fig. 2A). Western blot results showed that the expression of Occludin and Claudin-1 proteins in the duodenum of the DON group were decreased (*p* > 0.05) but not significantly compared with the control group. The expression of Claudin-1 was significantly reduced (*p* < 0.05), and the expression of Occludin was just a downward trend in the jejunum. Both Occludin and Claudin-1 expression in the ileum were significantly reduced (*p* < 0.05) (Fig. 2B). Immunohistochemistry showed that in the duodenum, ZO-1 and Occludin were mainly distributed on the free surface of epithelial cells, and Claudin-1 was mainly distributed in other cells except for goblet cells; in the jejunum, ZO-1 and Occludin were mainly distributed in the cytoplasm of other cells except for goblet cells, and Claudin-1 was mainly distributed around goblet cells and the free surface of epithelial cells; in the ileum, ZO-1 was mainly distributed on the basal surface of epithelial cells, Occludin was mainly distributed in the cytoplasm of epithelial cells, and Claudin-1 was only weakly distributed in the central chylous duct. The optical density results showed that compared with the control group, the expression of ZO-1, Occludin, and Claudin-1 in the duodenum and jejunum of the DON group were significantly decreased (*p* < 0.05), but the expression of ZO-1, Occludin, and Claudin-1 in the ileum were not significantly decreased (*p* > 0.05) (Fig. 2C and D). Taken together, it was demonstrated that the consumption of DON-contaminated feed inhibited the expression of ZO-1, Occludin, and Claudin-1 in the small intestine.

### The changes in microecology of cecal biota in broilers

**Fig. 3:**
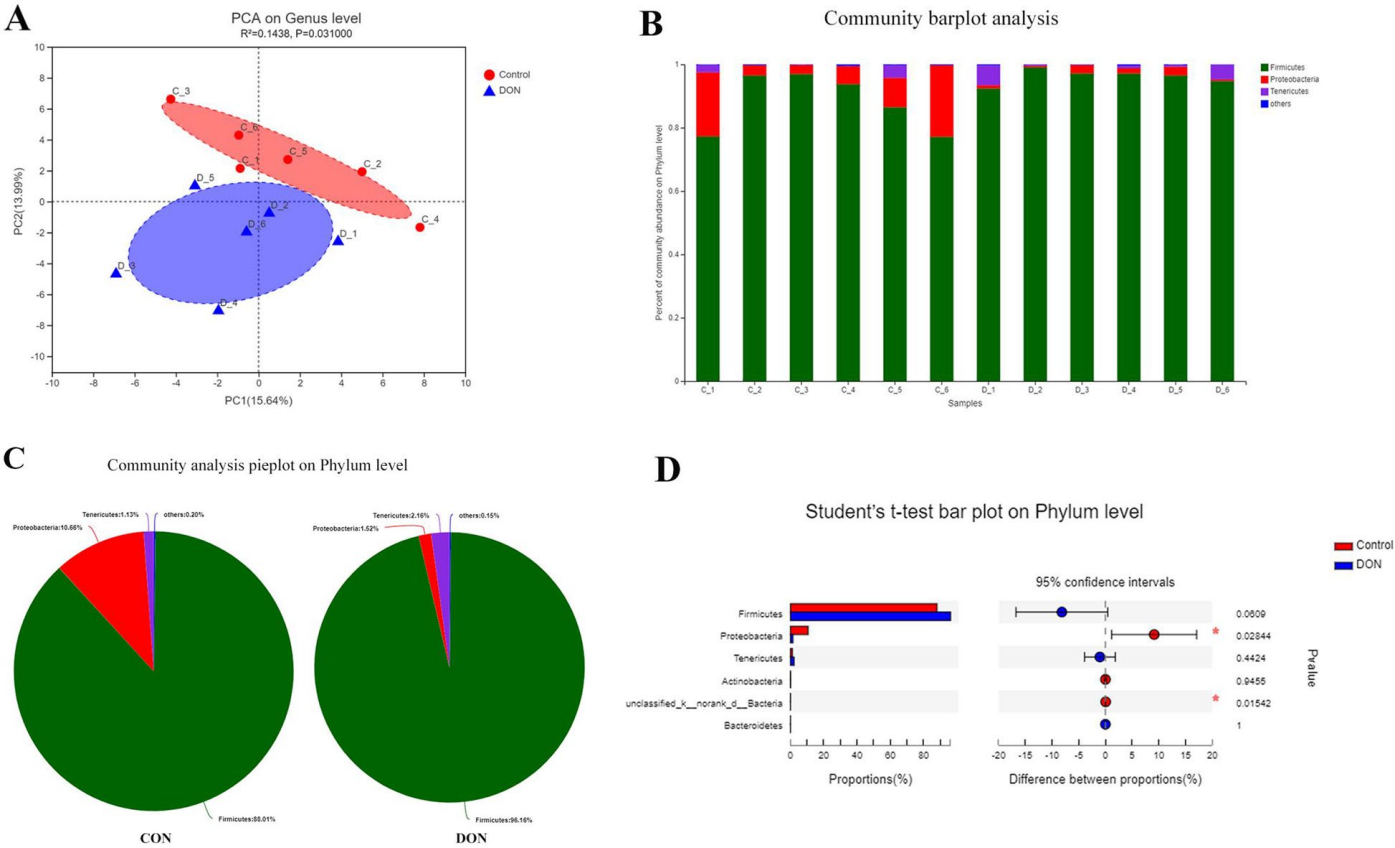
Microecological changes of caecal biota in broilers caused by DON. **A** The principal component analysis (PCA) of the cecum microbiota, R^2^ = 0.144, *P* = 0.031. **B**, **C** The diagrams of microbial community composition structure. **D** The significant difference analysis diagram between the DON group and CON group. *n* = 6 per treatment group

The PCA method in 16S rDNA high-throughput sequencing was used for principal component analysis. The results showed that the bacterial community composition of the control and the DON groups were significantly different. The dominant bacterial communities of the two groups were the same at the phylum level, but the relative abundance was different (Fig. 3A). At the phylum level, the dominant bacteria in the two groups were *Firmicutes*, *Proteobacteria*, *Tenericutes*, and unknown bacteria. The proportions of these mentioned bacteria at the phylum levels were 88.01%, 10.66%, 1.13% and 0.20% for control group, and 96.16%, 1.52%, 2.16%, and 0.15% for DON group, respectively (Fig. 3B, C). The analysis of differences between the CON and DON groups showed that the *Firmicutes*, *Tenericutes,* and *Bacteroides* in the DON group were increased at the phylum level, while *Actinomycetes*, *Proteobacteria*, and unknown bacteria were decreased, and *Proteobacteria* and unknown bacteria decreased significantly (*p* < 0.05), as shown in Fig. 3D.

## Discussion

DON is the most common member of the trichothecene group of mycotoxins. In recent years, DON-contaminated feed has caused great economic loss to the livestock industry. It is well-documented that DON-contaminated feed leads to reduced performance, feed intake, body weight gain, and feed conversion rate. High-dose acute DON exposure results in vomiting, diarrhea, and neurological symptoms in humans and animals [10]. After oral administration, DON passes through the body's small intestinal barrier and is rapidly absorbed. It destroys the small intestinal mucosal layer, damages villi, and causes epithelial shedding, which consequently affects the absorption of nutrients by mucosal epithelium [12, 13]. Therefore, the intestines are the primary target of DON attack [14], and the molecular mechanism involved in the influence of DON on the intestinal tract of broilers is very crucial for successful broiler production.

The small intestine is the main site for the transportation and absorption of nutrients in the body. The digestion, absorption, and function execution of the body are closely related to the small intestinal villi. The intact structure and good functions of the small intestinal villi are essential for better digestion and absorption of nutrients which leads to the healthy growth of the animal body [15]. The crypt between two villi is the site where the villi cells regenerate. The villus height to crypt depth ratio represents the secretory function of the small intestine [16]. The depth of the crypt reflects the rate of cell production and the shallower crypts indicate an increase in cell maturation rate and secretory function. Here, we found that the small intestinal villi of broilers fed with DON-contaminated feed were swollen, ulcerative, fractured, and the shorter villi reduced the absorption area of the intestinal tract, preventing the chyme from being in good contact with the intestinal tract and reducing the intestinal swing ability which resulted in an overall reduction both in digestion and absorption of nutrients in the intestine. The increase of the crypt depth indicated a decrease in the intestinal epithelial mature cell numbers, which reduced the function of upper small intestinal cells. A decrease in the ratio of villus height to crypt depth indicated that the regeneration and metabolism of small intestinal epithelial cells slows down. Therefore, DON destroyed the mechanical barrier of the small intestinal mucosa.

On the molecular level, the tight junction proteins between adjacent epithelial cells are the basic structure of small intestinal mucosa, which directly affects the intestinal barrier function [17]. Here, the distribution of tight junction protein ZO-1, Occludin and Claudin-1 were affected by DON in epithelial cells. From the duodenum to the ileum, ZO-1 and Occludin were differentially distributed from the free surface of epithelial cells to the basal surface of epithelial cells, with weaker expression caused by DON. Therefore, the barrier constructed by ZO-1 and Occlausin was destroyed as the manner of spatial moving by DON, which affected the protection function of epithelial cells. While the decreased Claudin-1 was weakly expressed in other cells except for goblet cells in the duodenum and mainly in goblet cells in jejunum and none in epithelial cells in the ileum, which suggested that Claudin-1 might play minor roles in the function of epithelial cells. Therefore, DON reduced the expression of ZO-1, Occludin, and Claudin-1 in the small intestine, especially in the duodenum and jejunum, which provided favorable conditions for pancreatic juice and bile to damage duodenal and jejunal epithelial cells and resulted in reduced broiler performance.

The digestive enzymes in the small intestine play important roles in the digestion and absorption of nutrients. The level of digestive enzymes in the small intestine directly reflects the ability of animal nutrient utilization [18, 19]. Amylases catalyze the hydrolysis of glycogen, produce maltose and glucose and provide energy for the body [20]. Trypsin breaks down protein and provides amino acids for the body [21]. Under the combined action of bile salts, lipases break down fat into glycerol, fatty acids, and monoglycerides, and thus provide energy for the body. Consumption of DON-contaminated feed reduced the amylase, trypsin, and lipase activities in the small intestine of broilers, impaired the digestion and absorption of nutrients in the small intestine, and resulted in the reduction of broilers' performance.

In addition, the broilers intestinal microbiota is of physiological importance in the host's health and production performance in terms of host nutrient absorption, immune barrier, detoxification, immune system development, and regulation [22]. Therefore, intestinal microbes are essential for stable production performance. As an active “organ” that interacts with the gastrointestinal environment, the biota provides nutrients and vitamins to the organism, transduces hormone information, and ultimately affects the main metabolic pathways [23, 24]. Bacterial groups dominate the microbial community inhabiting broilers [25]. In this research, we found that *Bacteroidetes*, *Firmicutes*, *Proteobacteria*, and *Tenericutes* were the main phyla in the broiler’s intestines, which was consistent intestine with the results of Oakley [26] and Qu [27]. Post consumption of the DON-contaminated feed, the *Firmicutes* and *Bacteroides* were increased. *Firmicutes* and *Bacteroides* are involved in energy regulation [7, 28, 29], fat metabolism [30, 31], and they participate in the body's absorption or energy storage [32], which might be the important factors to reduce the bodyweight of broilers. *Tenericutes* were reduced by DON, which revealed the environment of the cecum was disturbed to decrease the performance of the broiler. The *Proteobacteria* was increased, such as *E. coli*, *Salmonella*, etc., so we inferred the consequences of harmful bacteria following disruption of the microbiota's environment, which could lead the performance of broiler to be declined. The *Actinomycetes* can inhibit the growth of pathogenic bacteria [33], which was reduced by DON. Therefore, the microbiota homeostasis could be affected to decrease the growth performance.

## Conclusions

DON damaged the intestinal tract by destroying the morphology of the small intestinal villi, the tight junction protein of the small intestine, the enzyme activity of the intestine, and the biota of the caecum, leading to declined broiler’s performance.

## Materials and methods

### Reagents and antibodies

Amylase (C016-1–1), Trypsin (A080-2–2), and Lipase (A054-2–1) were purchased from Nanjing Jiancheng Bioengineering Institute (Nanjing, China). TRIzol reagent was obtained from Tiangen Biotech Co., Ltd. (Beijing, China). Reverse transcription kit from TaKaRa (Japan), 2 × SYBR Green qPCR Master Mix from Bimake (Houston, USA), RIPA cell lysate, protease inhibitor and phosphatase inhibitor were purchased from Solarbio (Beijing, China). β-actin Monoclonal Antibody was purchased from Immunoway Bitechnology company (Jiangsu, China). ZO-1(ab96587) was obtained from Abcam (Cambridge, MA, USA). Occludin (bs-10011R) was obtained from Bioss (Wuhan, China). Claudin-1 (13,050–1-AP) was obtained from Proteintech (Wuhan, China) and a DAB kit was purchased from CWBIO (Beijing, China). *Fusarium graminearum* ACCC 37,687 was provided by Professor Yang Xiaojun, College of Animal Science and Technology, Northwest A&F University. AA broilers were purchased from Shanxi Elephant Farming and animal husbandry group (China).

### Preparation of DON contaminated feed

*Fusarium graminearum* ACCC 37,687 was inoculated into potato dextrose agar medium and cultured at 27 °C for 7 d to obtain a solid culture of *Fusarium graminearum*, incubated into potato broth at 27 °C and 180 rpm for 7 d to obtain a liquid culture. 25 g solid culture and 100 mL liquid culture were mixed into 200 g rice, and cultured for another 28 d to obtain DON-contaminated fragrant rice. According to the previous reported method [10, 11], we detected the content of DON in the contaminated rice. The results showed that the content of DON was 22.62 mg/kg (Table 5). When indicated, the crushed DON contaminated rice was diluted in basal diet to achieve the required density.

**Table 5:** The content of toxins in the basal diet and contaminated rice

groups	Aflatoxin B2 μg/kg	Aflatoxin G1 μg/kg	Aflatoxin G2 μg/kg	DON mg/kg	Zearalenone μg/kg
Basal diet	< 0.01 (0)	< 0.03 (0)	< 0.01 (0)	0.20	< 5.0 (0)
Contaminated rice	< 0.2 (0)	< 0.3 (0.15)	< 0.3 (0)	22.62	< 5.0 (0)

## Animals and treatment

A total of 180 1-day-old AA broilers weighed 55.67 ± 1.43 g was purchased from Shanxi Elephant Agriculture and Animal Husbandry Group Co., Ltd, and randomly divided into two groups: the control group and the DON group.Ten broilers pens per group, with 9 broilers per pen. At the day for collecting samples, 2 broilers were randomly selected from each pen. Broilers in control group and DON group received a basal diet and 10 mg/kg DON contaminated basal diet, respectively. The ingredients and composition of the basal diets were listed in Table 6. All broilers were free drinking, immunized with routine vaccines, and had routinely feeding management. After 7 d of feeding, 20 broilers in each group were euthanized by severing jugular vessels. The experimental procedure involving animals in this study was approved by the Experimental Animal Ethics Committee of Shanxi Agricultural University (Taigu, China).

**Table 6:** Ingredients and nutrient composition of the basal diets

Ingredients	Percentage (%)	Nutrient composition	Percentage (%)
Corn	61.17	Metabolism energy, (MJ/kg)	12.97
Soybean meal	29.50	Crude protein (%)	20.8
Fishmeal	6.50	Available P (%)	0.45
DL-Met	0.19	Ca (%)	1.02
L-Lys•HCl	0.05	Lys (%)	1.20
Bone Meal	1.22	Met + Cys (%)	0.86
Sodium chloride	0.37		
Microelement and Vitamin Compound Premix	1.00		
Total	100		

Premix can provide per kilogram of basal diet, Vitamin A 9500 IU, Vitamin E 30 IU, Vitamin D3 62.5 μg, Vitamin K3 2.65 mg, Vitamin B1 2 mg, Vitamin B2 6 mg, Vitamin B12 0.025 mg, Biological Element C 0.0325 mg, folic acid 1.25 mg, pantothenic acid 12 mg, niacin 50 mg, copper 8 mg, zinc 80 mg, manganese 80 mg, iodine 0.35 mg, selenium 0.3 mg

### Intestinal injury score of broilers

According to the method of the previous studies [34, 35], the small intestine of broilers was scored by gross morphology and histomorphology.

### Scanning electron microscopy

The tissue samples were fixed in glutaraldehyde by conventional method [36] and observed under a scanning electron microscope (JEM-6490LV, JEOL, Japan).

### Histopathological observation of small intestine

The duodenum, jejunum, and ileum were fixed in Bouin’s solution for 24 h, tissues were sectioned, stained with H&E, and mounted with neutral gum. The histological changes in intestinal tissue were observed with a microscope. Image J software (National Institutes of Health, USA) was used to measure the villus height (V) and crypt depth (C), and the villi/crypt ratio (V/C) was calculated.

### Detection of small intestine tight junction proteins

The total RNA of the duodenum, jejunum, and ileum samples were extracted, qRT-PCR was used to detect the expression of ZO-1, Occludin, and Claudin-1 in each intestinal segment, and the 2^−△△ct^ method was used to calculate the relative expression of ZO-1, Occludin and Claudin-1 mRNA level [37–39]. The primers used for RT-qPCR are presented in Table 7. Western blot was performed to measure the proteins expression of the duodenum, jejunum, and ileum samples. In brief, the proteins were extracted using the RIPA lysate, and the protein concentration was detected using a BCA assay kit (Beyotime Biotechnology). Then 60 μg proteins from each sample were separated on 12% SDS-PAGE gel, which was then transferred onto a polyvinylidene fluoride (PVDF) membrane (Millipore, USA). The membrane was cut according to the molecular weight of target proteins and then was blocked with 5% nonfat milk for 2 h at room temperature and then incubated with primary antibodies against β-actin (1:5000), Occludin (1:2000), Claudin-1(1:2000) at 4℃ overnight and then with HRP-conjugated goat anti-mouse and goat anti-rabbit IgG for 2 h at room temperature, respectively. The membranes were subsequently washed with TBST three times, and the protein bands were detected with exposure to X-ray film using an eECL Western Blot Kit (CWbio Inc., China). The densitometric values of protein bands were quantified by using Image-Pro Plus 6.0. Immunohistochemical methods were used to detect the localization of ZO-1, Occludin, and Claudin-1 in the duodenum, jejunum, and ileum, the method was the same as the report by Zhao [40], The optical density was measured by Image-Pro Plus software.

**Table 7:** Primers of RT-qPCR

Gene name	Sense (5’-3’)	Antisense (5’-3’)
β-actin	ACCGCAAATGCTTCTA AACC	ATAAAGCCATGCCAAT CTCG
ZO-1	GTGGTGCTTCCAGTGCCAACAG	GCTTGCCAACCGTAGACCATAC
Occludin	CGCAGATGTCCAGCGGTTACT	CAGAGCAGGATGACGATGAGG
Claudin-1	CTGCTCTGCCTCATCTGCTTC	CCATCCGCCACGTTCTTCACC

### Determination of lipase, amylase, and trypsin of the small intestine

The collected duodenum, jejunum, and ileum were ground, and then the lipase, amylase, and trypsin of mucosal homogenates in the duodenum, jejunum, and ileum were measured using commercial kits (Jiancheng Bioengineering Institute, Nanjing, China) according to the manufacturer’s instructions.

### High-throughput sequencing of cecal intestinal biota

The 16S rDNA high-throughput sequencing method was used to detect the bacterial biota in the cecum by Majorbio Bio-Pharm Technology Co., Ltd. (Shanghai, China), the detailed steps referred to Chen [41].

### Growth performance

Broilers in both groups were fed for 7 d and weighed on fasting basis. The average daily gain (ADG, G), the average daily feed intake (ADFI, F) and F/G were calculated.

### Statistical analysis

All data were expressed as mean standard error of the mean (Mean SEM). The differences among groups were analyzed by t-test using Graphpad Prism 5 (Graphpad Software, USA), *p < 0.05, **p < 0.01, ***p < 0.001 were considered statistically significant.

## Supplementary Information

**Additional file 1: Fig. S1.** The original blots of Fig. 2B.

## Data Availability

The data that support the findings of this study are available on request from the corresponding author. The data are not publicly available due to privacy or ethical restrictions.

## Abbreviations

DON: Deoxynivalenol
DG: Average Daily Gain
ADFI: Average Daily Feed Intake
F/G: Ratio of feed to gain
