# Supplementary figures and images for: Deoxynivalenol damages the intestinal barrier and biota of the broiler chickens
Source: BMC Vet Res. 2022 Aug 15;18:311. doi: 10.1186/s12917-022-03392-4 (PMC9377127; doi:10.1186/s12917-022-03392-4)

Fig.S1 The original blots of Fig. 2B.

Duodenum:

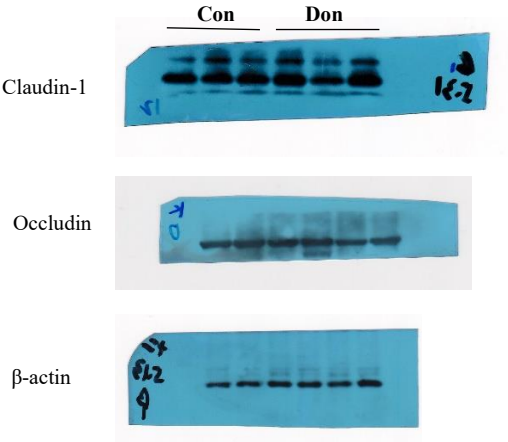

Jejunum:

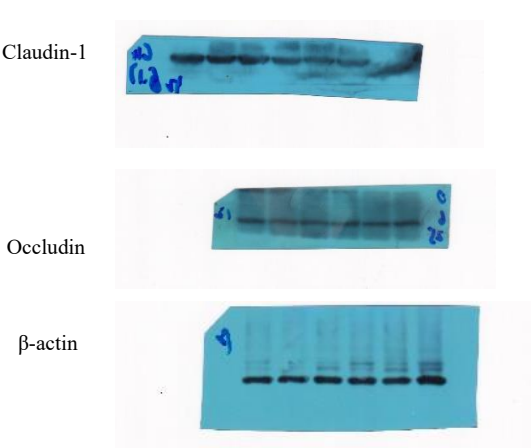

Ileum:

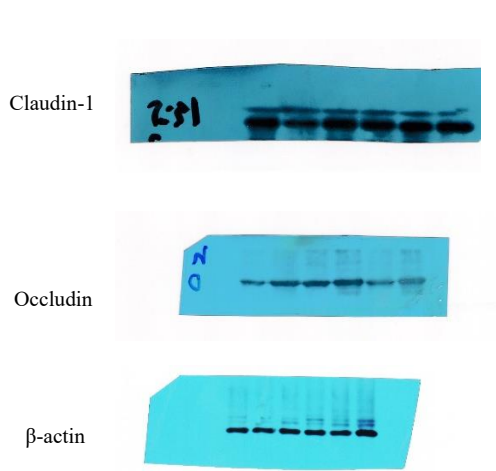

Supplement: Supplementary file 1 — Additional file 1: Fig. S1. The original blots of Fig. 2B. [file 12917_2022_3392_MOESM1_ESM.pdf]
